# How Does Neighborhood Environment Influence Dementia Onset?: Evidence From the National Health and Aging Trends Study

**DOI:** 10.1093/geroni/igaa057.205

**Published:** 2020-12-16

**Authors:** Yi Wang, Weidi Qin

**Affiliations:** 1 University of Iowa, Iowa City, Iowa, United States; 2 Case Western Reserve University, Cleveland, Ohio, United States

## Abstract

Existing research has established significant associations between older individuals’ social participation (e.g., visiting friends and family, going out for enjoyment, etc.) and their risk of developing dementia. However, little was known about the role of neighborhood environment (i.e., neighborhood social cohesion, neighborhood physical disorder). This study aims to investigate the moderating effects of neighborhood environment on the relationship between social participation and dementia incidence using data obtained from 8 waves (Rounds 1 through 8) of the National Aging and Trends Study. 7,416 Medicare beneficiaries aged 65 or above who were community-dwelling and free from dementia at Round 1 (2011) were selected. Among them, 1,005 participants developed dementia in the followed-up years from 2012 to 2018. We conducted survival analysis using the accelerated failure time (AFT) models to examine the moderating effects of neighborhood social cohesion and neighborhood physical disorder on the onset of dementia. The unit of time-to-event data was in years. Findings showed that the interaction term of neighborhood physical disorder and individual social participation was significant, indicating neighborhood physical disorder moderates the protective effects of social participation on dementia incidence. Specifically, among older adults who participate in social activities at the same level, the progression to the onset of dementia is 1.06 times faster for those living in neighborhoods with higher level (i.e., one unit increase in the score) of physical disorder. The findings recommend modification of physical environment at the neighborhood level to facilitate social participation for the betterment of cognitive health in later life.

